# Complete Genome Sequence of *Klebsiella variicola* Strain HKUOPLA, a Cellulose-Degrading Bacterium Isolated from Giant Panda Feces

**DOI:** 10.1128/genomeA.01200-15

**Published:** 2015-10-15

**Authors:** Matthew Guan-Xi Lu, Jingwei Jiang, Lirui Liu, Angel Po-Yee Ma, Frederick Chi-Ching Leung

**Affiliations:** a The Independent Schools Foundation Academy, Hong Kong SAR, China; b School of Biological Sciences, The University of Hong Kong, Hong Kong SAR, China; c Bioinformatics Centre, Nanjing Agricultural University, Nanjing, China

## Abstract

We report here the complete genome sequence of *Klebsiella variicola* strain HKUOPLA, isolated from a giant panda feces sample collected from Ocean Park, Hong Kong. The complete genome of this bacterium may contribute toward the discovery of efficient cellulose-degrading pathways.

## GENOME ANNOUNCEMENT

Strain HKUOPLA was originally determined by a functional assay with a decolorizing ring in carboxymethylcellulose (CMC)-Congo red and subsequent screening based on its ability to grow in minimal basal salt medium with Whatman filter paper grade 1 supplied as the sole carbon source under aerobic conditions (1). The strain was identified as *Klebsiella variicola* by comparing its sequence to the nucleotide database in NCBI using BLASTn, and the BLAST result showed the highest identity, 95%, to strain At-22 (NCBI accession no. CP001891) (2). The genus *Klebsiella* is facultative anaerobic, having both respiratory and fermentative type metabolisms. Most strains produce acid, gas, or 2,3-butanediol as a major end product of glucose fermentation (3).

The genomic DNA of strain HKUOPLA was extracted using the cetyltriammoniumbromide (CTAB) protocol (4) from a pure aerobic culture in Luria broth. Its quality and quantity were examined and measured using a NanoDrop 2000 spectrophotometer (Thermo Scientific) and the Quant-iT PicoGreen double-stranded DNA (dsDNA) kit (Invitrogen), respectively. A 2 × 300 MiSeq library and a MiSeq paired-end library were constructed and sequenced with the Illumina MiSeq at the Bioinformatics Centre, Nanjing Agricultural University, Nanjing, China. The Illumina MiSeq platform achieved 1,238,299 reads >250 bp, with a mean quality score of >30. All these reads were assembled, using the Newbler version 2.7 assembly software program (Roche), into 36 large contigs, with a 61-fold coverage of 5,062,551 bases. Gaps between contigs were closed by bioinformatics tools and subsequently assembled using the SeqMan software (DNAStar).

The complete genome of strain HKUOPLA was submitted to the NCBI Prokaryotic Genome Automatic Annotation Pipeline (PGAAP) for annotation.

The *K. variicola* strain HKUOPLA has one chromosome, which is 5,090,052 bp in size, with a G+C content of 58.04%. The genome has a total of 4,757 genes, 4,614 predicted coding sequences (CDSs), 31 pseudogenes, and 12 frameshifted genes. Some other features were identified, including 84 tRNAs, 25 rRNAs, and 3 noncoding RNAs (ncRNAs).

### Nucleotide sequence accession number.

The complete genome sequence of *K. variicola* strain HKUOPLA has been deposited in GenBank under the accession no. CP012252 for the chromosome. The version described in this study is the first version.
